# Reproductive Outcomes Following Robot-Assisted Laparoscopic Myomectomy: 10 Years’ Experience

**DOI:** 10.7759/cureus.70232

**Published:** 2024-09-26

**Authors:** Rooma Sinha, Bana Rupa, Rohit Raina, Moumita Bag, Deepika HK, Padmapriya Reddy

**Affiliations:** 1 Gynecology, Apollo Health City, Hyderabad, IND

**Keywords:** fertility, fibroids, myomectomy, pregnancy, robotic-assisted laparoscopic myomectomy

## Abstract

Introduction

Robotic-assisted laparoscopic myomectomy (RALM) is being increasingly performed for large and multiple uterine myomas because of precise surgery and offers many short-term benefits over conventional surgeries; however, long-term effects in terms of reproductive outcome are not studied well. Our study aims at finding the pregnancy rate after RALM. We also studied secondary outcomes (rate of live births after RALM, conception technique (spontaneous or in-vitro fertilization (IVF)), and the correlation between pregnancy outcome and size, position, and number of fibroids removed.

Methods and materials

A single-center, retrospective observational study was conducted with patients who underwent RALM for fertility enhancement from August 2012 to May 2023 in the Department of Gynecology to know fertility outcomes. The RALM was performed using da Vinci Si and Xi.

Results

Out of 243 cases of RALM, 114 cases were operated for fertility enhancement (21 were lost to follow-up, and seven were not in the conception period). The outcome was analyzed for 86 patients (54 primary and 32 secondary infertility). The mean age was 31.99 ±4.53 years, and the mean fibroid weight was 347.83 ± 259.72 g. Forty-nine cases conceived spontaneously (six abortions) and 37 cases by IVF (12 failed, five abortions). The mean time to conception was 13.95 ± 4.82 months. All 63 cases were delivered by cesarean section (44 cases at 37-38 weeks, 17 cases at 36 weeks due to associated comorbidities, and two twin pregnancies delivered at 35 weeks). The pregnancy rate was 74/86 (86.04%) and the live births rate was 63/86 (73.25%). No statistical difference was found regarding the number of fibroids removed (p = 0.570) and size (p = 0.285) with pregnancy outcome. None of the myoma characteristics were related to abortion.

Conclusion

In our study, RALM was found to be an effective method for fertility enhancement.

## Introduction

Myomectomy is the gold standard treatment for symptomatic fibroids who desire fertility preservation. Robotic-assisted surgery has seen rapid development and integration in gynecology, considering its several advantages over conventional laparoscopy because of precise surgery with three-dimensional imaging (3D imaging) and meticulous suturing even in cases of larger myomas [1].

Short-term benefits offered by a robotic platform in myomectomy are less intraoperative bleeding, less postoperative pain, shorter hospital stay, and faster postoperative recovery [2]. Limited data are available describing fertility outcomes after robotic-assisted laparoscopic myomectomy (RALM) with a wide range of pregnancy rates of 33-77.8% [1,3-7].

In this study, we studied long-term benefits in terms of reproductive outcomes with a focus on the live birth rate after RALM, conception technique (spontaneous or in-vitro fertilization (IVF)), and the correlation of the pregnancy outcome with size, position, and number of fibroids removed.

## Materials and methods

We performed a single-center, retrospective observational study in patients who underwent RALM by a single surgeon for fertility enhancement from August 2012 to May 2023 in the Department of Gynecology in Apollo Health City, a tertiary care center in Hyderabad, India, to know fertility outcomes. We included females, with primary or secondary infertility, looking for conception in which other causes of infertility were ruled out. Any previous surgery done for myoma or associated endometriosis cases were excluded from the study.

**Figure 1:**
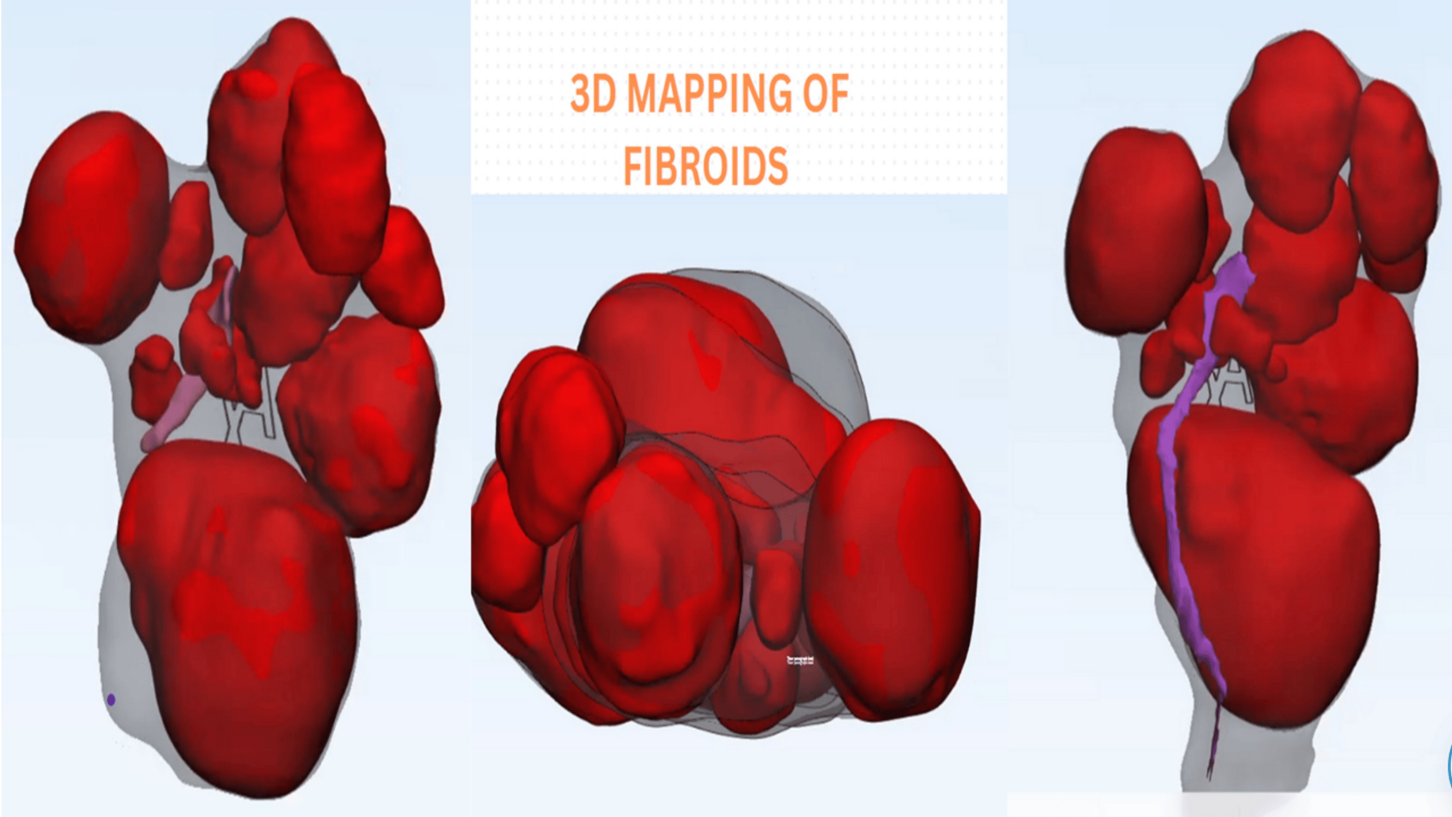
Three-dimensional (3D) mapping of fibroids

The RALM was performed using da Vinci Si and Xi (Intuitive Surgical, Sunnyvale, CA). For all patients, a preoperative myoma mapping (to know the number, size, and location) was done using magnetic resonance imaging (MRI), and in the last two years, we also added 3D mapping (material mimic software) (Figure 1) in cases of large and difficult cases.

Preoperative anemia correction was done (iron infusion, blood transfusion, or oral iron tablets). For intraoperative hemorrhage control inj. vasopressin (diluted 20 units in 200 ml NS) and tablet misoprostol (400 mcg) per rectally were used.

**Figure 2:**
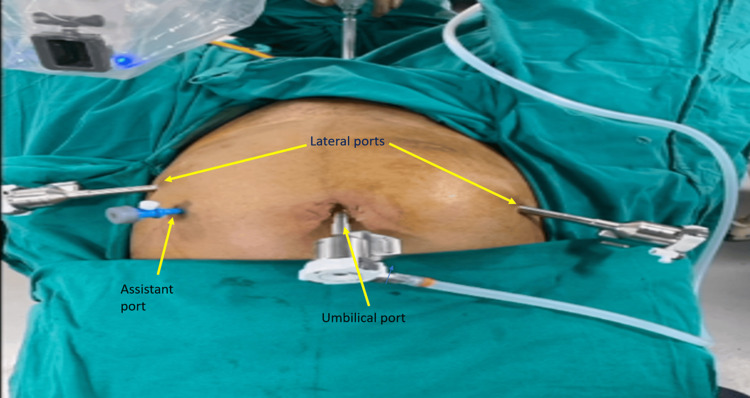
Port placement during robotic-assisted laparoscopic myomectomy (RALM)

In both surgical systems, we followed the “two arms, three instrument technique” using curved monopolar scissors and fenestrated bipolar forceps for dissection and vessel sealing, respectively, and a mega needle holder for suturing. A 5 mm laparoscopic port was used for assistance. RUMI (CooperSurgical, Connecticut, USA) uterine manipulator was used for manipulation (Figure 2).

We used fenestrated bipolar forceps @ effect 3, 60-watt setting and curved monopolar scissors @ effect 3, and 100-watt setting (ERBE VIO dV 2.0). Uterine closure was done using barbed suture (V-loc (Covidien) no. 0) in the multilayer depending on the depth of the incision or defect created. Intraoperatively, methylene blue dye was injected in the uterine cavity to identify any breach in the endometrial cavity. No adhesion prevention barrier was used. Finally, myomas were retrieved using cold knife morcellation in an Endo bag through an umbilical incision. Patients were advised for conception after six months. Patients who conceived spontaneously and by IVF post-RALM were included in the study.

Statistical analysis

Hospital records were retrieved of the cases as per our inclusion criteria, and demographic details, clinical history (primary or secondary infertility), fibroid location, grade and surgical details regarding size and number of fibroids removed, endometrial breach, operative time, blood loss, conversions, and any reported complications were tabulated. We were not their primary obstetrician or IVF specialist, so details of their reproductive procedure (IVF or spontaneous conception), outcomes (success and failure), and mode of delivery (cesarean section or vaginal delivery) were confirmed through telephonic interviews.

Ethical approval

Institutional Ethics Committee of Biomedical Research of Apollo Hospitals, Hyderabad, issued approval (IRB: AHJ-C-S-012/09-22), dated 29-09-2023.

## Results

**Figure 3:**
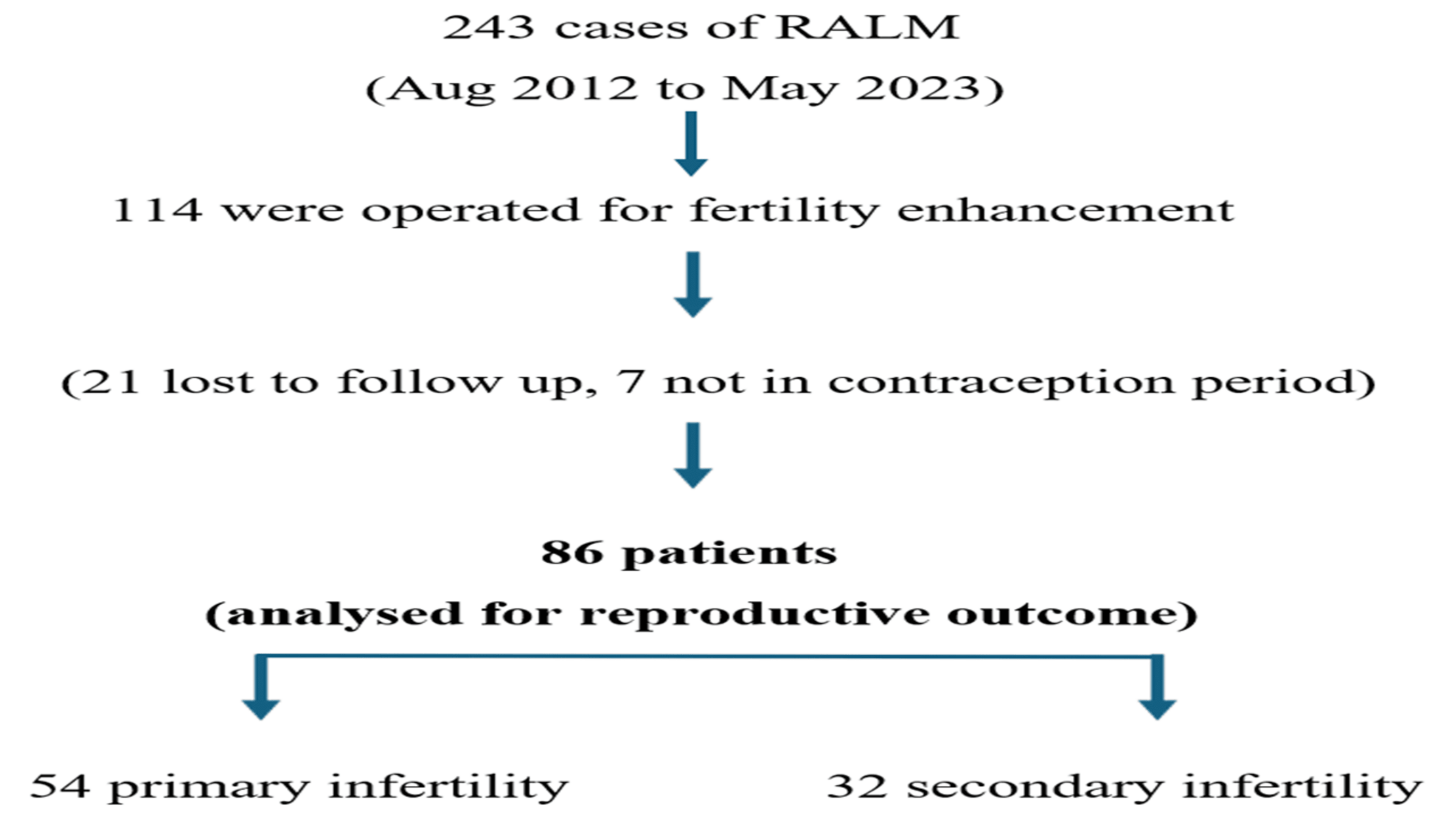
Flow chart depicting the case profile

We had a total of 243 cases of RALM, out of which 114 cases were operated for fertility enhancement (21 were lost to follow-up and seven were not in the conception period). Hence, reproductive outcome following RALM was analyzed for 86 patients (54 primary infertility and 32 secondary infertility) (Figure 3).

In our study, the mean age was 31.99 ±4.53 years, and the mean BMI was 27.60 ± 3.99 kg/m^2^. The most common location was the anterior wall fibroid at 41.9% (36/86), and the most common grade of fibroid was FIGO 4 (36%), followed by FIGO 5. The mean fibroid weight was 347.83 ± 259.72 g with a mean fibroid size of 8.49 ± 2. 57 cm (≥8 cm in 70.93%). The mean number of fibroids removed in the study was 2.10 ± 1.81 (≥3 in 29.07%). We observed endometrial breach in 13.15% of the cases. The mean operative time was 147.33 ± 58.49 min, with a mean blood loss of 110.47 ± 70.41 ml. None of the RALM resulted in a conversion to laparotomy and any major surgical complications (Tables 1, 2).

**Table 1:** Demographic characteristics *SD: standard deviation

Patient characteristics (n = 86)	n (%)	Mean	SD^*^
Age (years)		31.99	4.53
BMI (Kg/m^2^)		27.60	3.99
Primary infertility	54 (62.79%)		
Secondary infertility	32 (37.21%)		

**Table 2:** Surgical characteristics *SD: standard deviation; †FIGO: International Federation of Gynaecology and Obstetrics

Surgical characteristics	n(%)	Mean	SD^*^
Location of fibroid			
Anterior	36 (41.86%)		
Posterior	19 (22.09%)		
Lateral	11 (12.79%)		
Fundal	20 (23.26%)		
Grade of fibroid (FIGO^†^)			
FIGO 4	36%		
FIGO 5	33.7%		
Operative time (mins)		147.33	58.49
Blood loss (ml)		110.47	70.41
Number of fibroids removed		2.10	1.81
Fibroid weight (g)		347.83	259.72
Fibroid size (cm)		8.49	2.57
Endometrial breach	13.15%		
Conversion	0		
Complications	0		

The post-surgery mean time to conception was 13.95 ± 4.82 months. Forty-nine cases (56.97%) conceived spontaneously (six abortions) and 37 cases (43.02%) by IVF (12 failed, five abortions) (Table 3). We did observe an abortion rate of 12.79%. All the cases who got pregnant were delivered by a caesarean section. Forty-four cases (69%) got delivered at 37-38 weeks, 17 cases (26.9%) at 36 weeks due to associated comorbidities (pregnancy-induced hypertension, obstetric cholestasis, and gestational diabetes mellitus), and two cases (3.1%) had twin pregnancy (IVF) delivered at 35 weeks. No case of uterine rupture was seen in our study.

**Table 3:** Fertility outcome data *SD: standard deviation

Fertility outcome	n (%)	Mean	SD^*^
Time to conception (months)		13.95	4.82
Conception			
Spontaneous	49 (56.97%)		
IVF	37 (43.02%)		

The pregnancy rate in our study was 74/86 (86.04%), and the live birth rate was 63/86 (73.25%). Three of our cases got delivered a second time also. However, no statistical difference was found with regard to the size of the fibroid (p = 0.285) and the number of fibroids removed (p = 0.570) with pregnancy outcome. None of the myoma characteristics were related to abortion.

## Discussion

During RALM, we used mainly curved monopolar scissors for sharp dissection, and wherever electrosurgery was required, only short bursts were used. Suturing was done using V-loc no. 0 (barbed suture) in multilayers for uterine reconstruction, maintaining a good hemostasis and uniform tension line for good apposition.

In our study, the majority of cases had fibroid size of ≥8 cm (70.93%), the number of fibroids removed was ≥3 in 29.07%, and we observed endometrial breach in 13.15% of the cases.

Pistofidis G et al. (2012) reported mostly subserosal or pedunculated fibroids (85.7%) and most of the fibroids ≤5 cm (71.4%). The study highlighted with minimal use of diathermy for hemostasis and multiple-layer suturing for repairing the myometrial defect in cases of intramural and subserosal myomas with deep intrusion to minimize uterine rupture [8].

The post-myomectomy time interval between pregnancy is unclear, and no specific guidelines exist. Francois Margueritte et al. in a systematic review found that most studies regarding lap and open myomectomy have a 12-36-month interval, while robotic surgery studies have reduced the interval from three to six months with a 2.9% uterine rupture vs. 75.7% in lap cases. However, no linear relationship was found between gestational age at the uterine rupture and the time interval from myomectomy to conception (p = 0.706), suggesting insufficient data to advise a minimal time interval between myomectomy and conception [9].

Huberlant et al. studied fertility outcomes after RALM. It showed a clinical pregnancy rate of 52.8% with a live birth rate of 41.5%. A cesarean section was performed in 17 cases (70.8%) and seven vaginal (29.1%) and found no cases of uterine rupture, despite a 15.1% endometrial breach [10].

Zhang Y et al. had a pregnancy rate of 49.6% in the laparoscopic group (p = 0.397), and the live birth rate was 33.2% in the laparoscopic group (p = 0.520). The study found that the locations of myomas did influence the live birth rate (34.0% in anterior myomas, 44.2% in posterior myomas, and 21.8% in others. The study concluded a 12-month interval after myomectomy after finding a shorter average interval in the live birth group [11].

J Claeys et al. in a systematic review reported a low risk of uterine rupture after myomectomy (0.75 %) and concluded available evidence did not discourage attempts for a trail of normal delivery [12].

Our study included complex cases in terms of size and number of fibroids, but it still showed a mean time to conception of 14.62 ± 5.50 months with advice for conception after six months and no uterine rupture noted. We had all cesarean sections as we were not the primary obstetrician for these cases, and we do suggest a trial of labor can be advised in low-risk obstetric cases.

Lu B et al. in a retrospective study of laparoscopic myomectomy had a pregnancy rate of 74.4%. They found that the greater the size of the fibroids removed, the lower the postoperative pregnancy rate (p-value = 0.011). However, no statistical significance was found with the age of the patient, number of fibroids, and type of fibroids with the pregnancy rate [13]. In our study, we found no relation between the size of the fibroid (p = 0.285) and the number of myomas (p = 0.570). The robotic arms easily allow surgeons to reach deeper places and space-limited areas and do a thorough repair, which is not easy using stick-like laparoscopic instruments. 

Morales HSG et al. compared open, laparoscopic, and robotic (n = 24) myomectomy in terms of reproductive outcomes. The pregnancy rates were 23.8% for open, 33.3% for laparoscopic, and 41.6% for robotic-assisted myomectomy. They found a higher number of live births after robotic myomectomy, even though not statistically significant (p = 0.744). The study highlighted the influence of the number of fibroids removed on spontaneous pregnancy, the pregnancy rate decreased with six or more leiomyomas removed (35% in less than six fibroids versus 29% in more than six fibroids), which may even influence the absence of a prior pregnancy, and the achievement of post-surgery pregnancy [14].

Goldberg HR et al. in a retrospective study found a pregnancy rate of 70.0% (42/60) after RALM. They found a younger cohort of patients who became pregnant than those who did not become pregnant. However, no significant difference between groups with regard to BMI, myoma size, or intramural localization was seen [15].

Kang SY et al. conducted a retrospective study after RALM, 75.0% became pregnant. Six (85.7%) of the seven women in which no breach was seen were pregnant, and three (60%) of the five women with cases in which endometrium had been exposed during surgery became pregnant [16].

In our study, 56.97% conceived spontaneously and about 13.15% had an endometrial breach. We did not find any relation with the endometrial breach, which in turn highlights the importance of effective suturing in multilayers and minimal use of electrosurgery [5]. We observed an abortion rate of 12.79%, which is less than other studies [17]. Asmar J et al. in their study about robotic myomectomy suggested long-term results in terms of pregnancy outcomes showed an 80% pregnancy rate and 60% live rate [18].

Cela V et al. in their study had an overall pregnancy rate of 77.8% with a median time from surgery to conception of 16 months. In none of the cases was cavity breached. They closed uterine incisions in a single layer in 28.5% and double layers in 71.5%, and no uterine rupture was seen. This study had a very short sample size but clearly emphasized over the uterine closure technique and less use of electrocautery. They had spontaneous vaginal delivery in 28.6% and an elective cesarean section rate of 71.4% [5].

**Figure 4:**
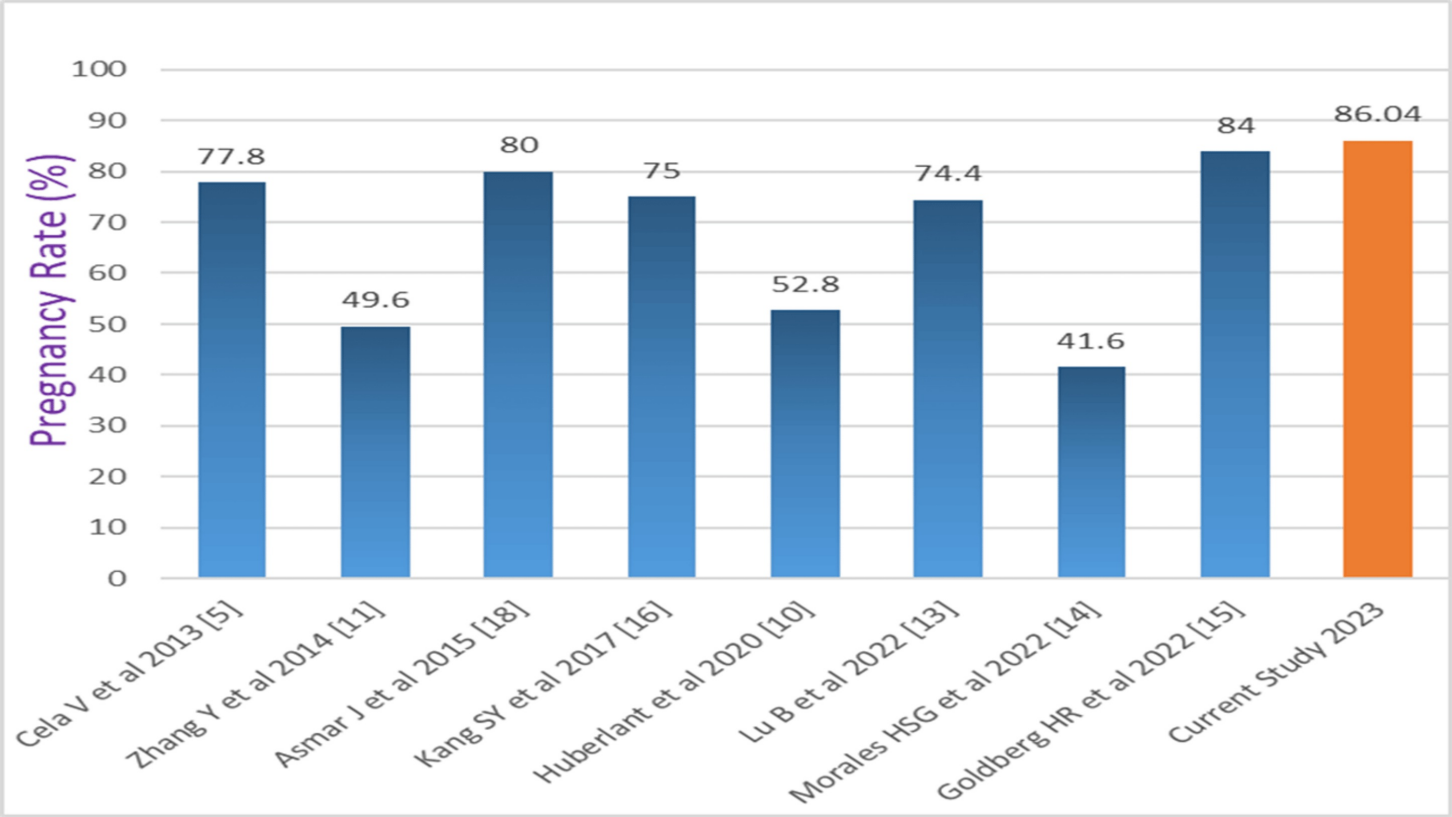
Comparison of the pregnancy rate (%) of our study with the literature

**Figure 5:**
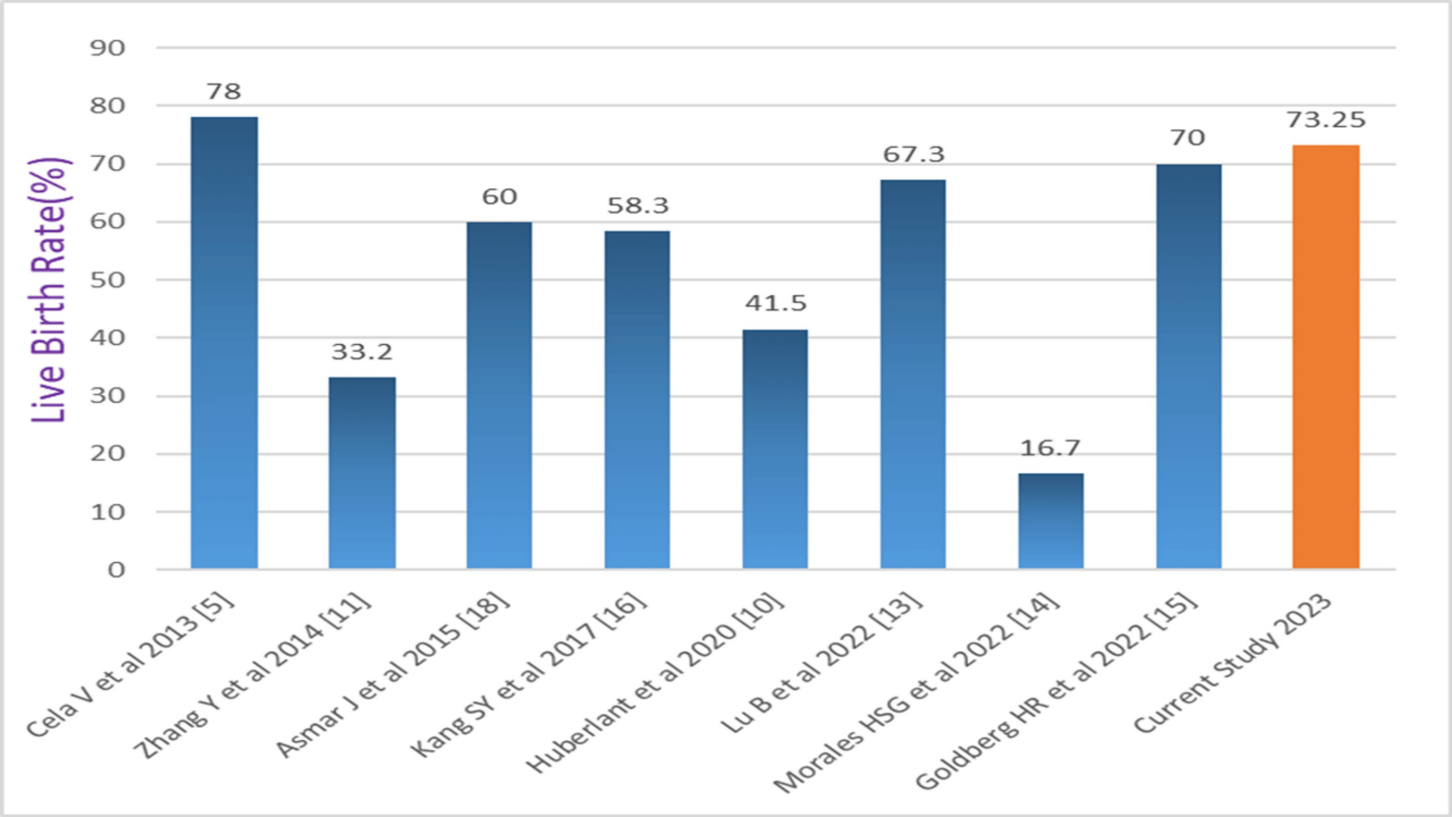
Comparison of the live birth rate (%) of our study with the literature

In our study, we had a pregnancy rate of 86.04% (74/86) and a live birth rate of 73.25% (63/86), which is comparable to other studies (Figures 4, 5).

Preoperative planning was done in our cases using MRI or 3D mapping helps in precise incision-reducing trauma to the myometrium and inadvertent breach to the endometrial cavity, which is also helped by robotic 3D vision. Intraoperative strategies including multi-layered uterine closure, avoidance of exposure to the endometrial cavity, and avoidance of excessive electro-cauterization to reduce devascularization are some of the important steps to achieve a perfect uterine reconstruction so that minimal complications are seen during future pregnancies.

Strengths and weakness

This is the first study from an Indian subcontinent about RALM and reproductive outcomes with a large sample size and 10 years of follow-up. The limitation of the study is its single-center, single-surgeon-based, and retrospective nature.

## Conclusions

In our study, a pregnancy rate of 86.04% was seen, which is similar to other previous studies and is comparable to other modalities of myomectomy, with no incidence of complications. Thus, RALM can be considered an effective method for fertility enhancement. However, we also find a need for a prospective multicenter trial to further evaluate the effectiveness and safety of RALM in terms of pregnancy outcomes.
